# Genetic Diversity of HIV-1 in Krasnoyarsk Krai: Area with High Levels of HIV-1 Recombination in Russia

**DOI:** 10.1155/2020/9057541

**Published:** 2020-09-10

**Authors:** Lada V. Maksimenko, Aleksey V. Totmenin, Mariya P. Gashnikova, Ekaterina M. Astakhova, Sergey E. Skudarnov, Tatyana S. Ostapova, Svetlana V. Yaschenko, Ivan O. Meshkov, Evgeniy F. Bocharov, Rinat А. Maksyutov, Natalya M. Gashnikova

**Affiliations:** ^1^State Research Center of Virology and Biotechnology Vector, Koltsovo 630559, Russia; ^2^Krasnoyarsk Regional Center for Prevention and Control of AIDS, Krasnoyarsk 660049, Russia; ^3^Novosibirsk Tuberculosis Research Institute, Novosibirsk 630040, Russia

## Abstract

More than a quarter of HIV-infected individuals registered in Russia live in Siberia. Unlike Central Russia where HIV-1 subtype A6 is predominant, in most Siberian regions since 2012, a new HIV-1 CRF63_02A1 genetic variant has spread, with the share of this variant attaining 75–85% among newly identified HIV cases. Krasnoyarsk Krai is considered to be a high-risk territory according to morbidity rate and HIV infection incidence among the population. The current paper aims to study the molecular epidemiologic characteristics of HIV-1 spreading in Krasnoyarsk Krai. Phylogenetic and recombination analyses of *pol* (PR-RT, IN) and *env* regions of the virus were used for genotyping 159 HIV-1 isolated in Krasnoyarsk Krai. 57.2% of the isolates belonged to subtype A (A6) specific to Russia, 12.6% to CRF63_02A1, and 0.6% to CRF02_AG_СА_, and in 29.6% HIV-1 URFs were detected, including URF63/А (23.9%), URFА/В (4.4%), and URF02/А (1.3%). In 6 of 7, HIV-1 URFА/В identical recombination model was detected; the origin of 38 URF63/А was proven to be the result of individual recombination events. Since 2015, a share of the population with newly diagnosed HIV who were infected with HIV-1 URF reached an exceptionally high rate of 38.6%. As distinct from adjacent Siberian regions, the HIV-1 CRF63_02A1 prevalence rate in Krasnoyarsk Krai is within 16%; however, the increased contribution of new HIV-1 into the regional epidemic development was observed due to the recombination of viruses of subtypes А, В, and CRF63_02A1. The difference between the described molecular epidemiologic picture in Krasnoyarsk Krai and in adjacent areas is likely caused by differences in predominant routes of HIV transmission and by more recent HIV-1 CRF63_02A1 transmission in the PWID group, which had a high prevalence of HIV-1 subtype A by the time of the new virus transmission, resulting in increased possibility of coinfection with various HIV-1 genetic variants.

## 1. Introduction

**Figure 1:**
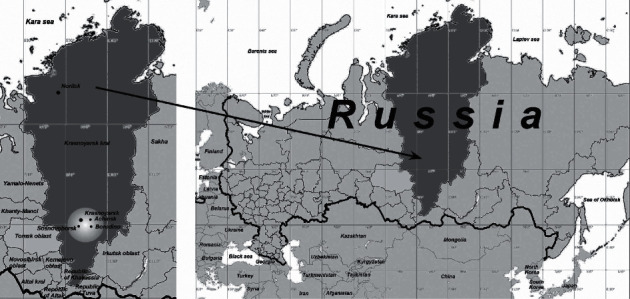
Geographic location of the Krasnoyarsk Krai. The geographic location of the Krasnoyarsk Krai on the right (highlighted in dark gray) and adjacent areas: Kemerovo Oblast, Novosibirsk Oblast, Tomsk Oblast, Altai Krai, and Irkutsk Oblast. Cities of Krasnoyarsk Krai (Krasnoyarsk and Norilsk) are highlighted, as well as the territory (light circle) where circulating HIV-1 was studied (left).

HIV infection epidemic in Russia continues to evolve [1], and HIV infection cases have been registered in all territorial entities of the Russian Federation. The number of Russian regions with a high prevalence of HIV infection (more than 0.5% of population size) reached 34 in 2018. Siberia (Siberian Federal District, SFD) significantly contributes to epidemic development, where more than a quarter of HIV-infected population of Russia is registered. Five SFD regions make the list of top 10 Russian territories with the highest HIV infection rates. In 2018, Krasnoyarsk Krai ranks 8^th^ in Russia and 5^th^ in SFD according to this index [2, 3], with adjacent Kemerovo Oblast, Irkutsk Oblast, Novosibirsk Oblast, and Tomsk Oblast being on the top of the list (Figure 1).

**Figure 2:**
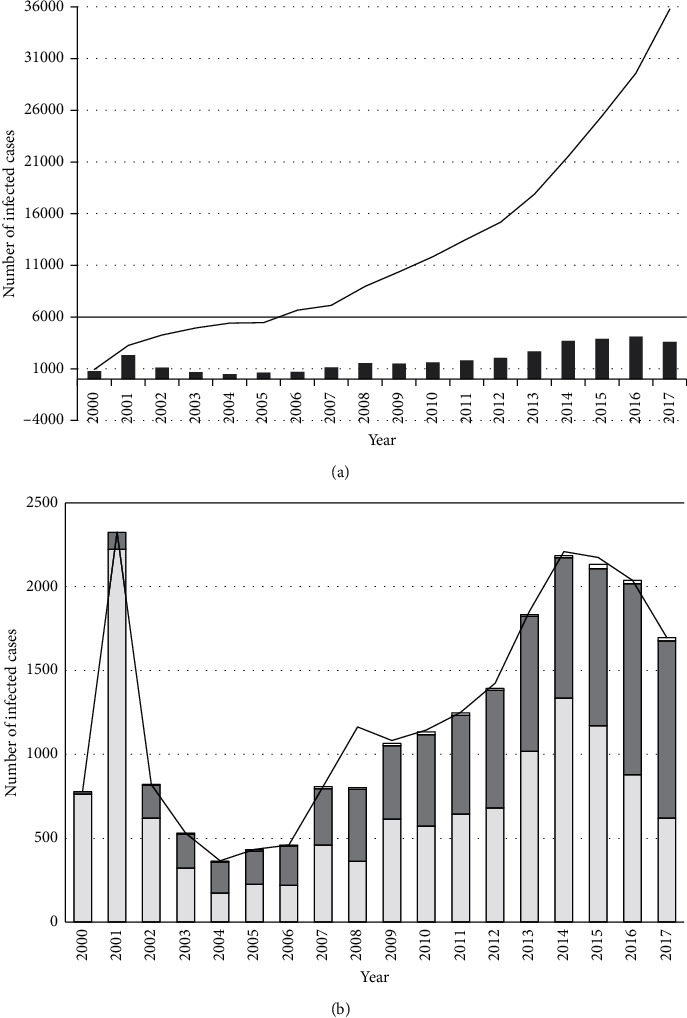
(a) Dynamics of HIV infection distribution in Krasnoyarsk Krai. The curve represents the total amount of registered HIV-infected individuals in Krasnoyarsk Krai; columns denote the number of new HIV infection cases by year. (b) Change of a share of HIV infection separate routes within the total amount of newly diagnosed patients with the detected route of infection by year. Sexual route of infection (HS) is highlighted in dark gray, people who inject drugs (PWID) are highlighted in light gray, and black denotes other HIV infection routes (vertical, homosexual, and hospital infection). The curve represents a change of the annual amount of newly diagnosed persons with the detected HIV infection route.

Krasnoyarsk Krai is the second largest federal subject with an area of 2 339 700 km^2^ and 2 876 497 residents, with the urban population accounting for 77.4%. Approximately 80% of the population lives south of Angara on one-tenth of the region area [4]. As reported by Krasnoyarsk Regional Center for Prevention and Control of AIDS, the first HIV infection in the region was officially registered in 1989. Before 1999, sporadic HIV infection cases were identified, and in 1999 HIV infection was detected in 156 people in 14 territorial entities of the region; this number was 3-fold higher as compared to the total number of identified HIV-infected people during ten preceding years. Over the following two years, the manifold increase in the number of newly detected HIV-infected patients was registered as well (Figure 2). The period from 2002 to 2006 can be considered as a time of epidemic process stabilization, and beginning from 2007 up until 2017 annual increase in registration of new cases had been observed again.

As in the case of other Russian regions, from the late 1990s onwards Krasnoyarsk Krai was demonstrating a sharp increase in the number of people who inject drugs (PWID) resulting from the beginning of large volume of opioids delivery to Russian Federation from Afghanistan through Central Asia countries (so-called North Route). At the same time, the first HIV infection cases were registered among PWID. Common for that time practice of risk behavior (shared use of syringes/needles) led to the fast spreading of HIV among PWID [5, 6].

In 1999–2001, a share of PWID among newly identified incidents of HIV infection in Krasnoyarsk Krai exceeded 95%. From 2002, Krasnoyarsk Krai has shown a decrease in the rate of HIV spreading among PWID; at the same time, PWID sexual partners became involved in the development of the epidemic process and this was reflected by the increase of heterosexual (HS) transmission route contribution in HIV spreading.

In 2004–2006, roughly equivalent numbers of HIV-1 infections were registered among PWID and HS (Figure 2). In 2007-2008, in adjacent Kemerovo Oblast and Novosibirsk Oblast, the second period of 3- to 4-fold increase in the number of new HIV infection cases among PWID was observed affecting the situation in Krasnoyarsk Krai. In 2007-2008, an increase in morbidity rate was observed, with the following relative stabilization but with higher quantitative indices of HIV distribution [7]. In 2013-2014, an epidemic outbreak in adjacent Tomsk Oblast was observed with 6- to 10-fold increase in newly registered HIV infection cases in the PWID group; Kemerovo Oblast, Novosibirsk Oblast, Omsk Oblast, Altai Krai, and Krasnoyarsk Krai demonstrated an increase in morbidity rates [8]. Starting in 2010, territory epidemics expansion in SFD has been caused by the beginning of synthetic drugs spreading [9], switching from opioid drugs to the new psychoactive substances (the so-called “synthetics” and “salts”) among PWID, and the involvement of large amount of population into the use of such drugs.

In total, 35 779 HIV-infected patients were registered in Krasnoyarsk Krai, and as of 30.06.2018, the HIV infection rate was 953.5 per 100 000 population. The cities of Krasnoyarsk and Norilsk demonstrate the highest HIV incidence rate. Men were predominant among the HIV-infected population in Krasnoyarsk Krai for the duration of the epidemic. A share of men among PWID has been stably registered at the level of 75–78%. In 2004–2007, two-thirds of detected HIV cases among people infected via heterosexual contacts (HS) account for women. Beginning from 2008, the gradual growth of a share of men infected via HS is registered: in 2016, the number of men in that group was equal to the number of women infected via HS. In recent years, 30- to 50-year-old people have mainly contributed to the HIV infection distribution in Krasnoyarsk Krai; in 2018, their number was 71.7% among newly detected HIV-positive patients, of which 60.5% were infected via HS and 36.5% were PWID.

Studies by Bobkov et al. demonstrated that epidemic development in Russia, including SFD, in the late 1990s and early 2000s, was caused by HIV-1 subtype A (A6) distribution among PWID [10–13].

As distinct from the European part of Russia, Urals, and Far East with currently predominant HIV-1 subtype A specific to Russia [14–20], after 2010–2014 in Novosibirsk Oblast, Tomsk Oblast, Kemerovo Oblast, and Altai Krai, 85% of newly detected HIV infections were caused by circulating recombinant form (CRF) HIV-1 63_02A1 [7, 8, 21, 22]. Furthermore, according to several studies, in Irkutsk Oblast up to 2012 and in Krasnoyarsk Krai up to 2008 exclusively Russian HIV-1 subtype A (A6) was detected [23, 24].

We proposed that the activation of HIV infection distribution in Krasnoyarsk Krai in 2013–2016 may have contributed to changes in the genetic variability of circulating HIV strains in that territory. This study aims to analyze the characteristics of current HIV-1 distribution in Krasnoyarsk Krai.

## 2. Materials and Methods

### 2.1. Ethical Approval

The study was approved by the Local Ethical Committee of State Budgetary Healthcare Institution of Novosibirsk Oblast “Municipal Infectious Diseases Hospital No. 1” (protocols no. 1 of March 28, 2017). Peripheral blood of the HIV-infected individuals was sampled by the medical staff of the “Krasnoyarsk Regional Center for Prevention and Control of AIDS,” with pretest and posttest psychological consultations. Epidemiologic data were collected by a professional epidemiologist from individuals under the study after they were acquainted with the project goals.

### 2.2. Study Participants and Sample Collection

From September to December 2017, 162 peripheral blood plasma samples were collected from HIV-infected patients in Krasnoyarsk Krai living in Krasnoyarsk (132), Norilsk (12), and other communities of the South of Krasnoyarsk Krai (18) who sought assistance in Krasnoyarsk Regional Center for Prevention and Control of AIDS. Plasma was separated within 3 hours after collection and frozen at −80°C for further use. 125 Krasnoyarsk patients were ART-naïve; 37 patients took APBT medication. The blood samples were linked with demographic and clinical data via coded ID numbers according to the requirements of medical ethics in Russia. The recorded characteristics for patients included their gender, age, most probable route of transmission, dates of the last negative and first positive tests for HIV, drug use, viral load, and CD4 cell count.

### 2.3. Quantification of Plasma HIV-1 RNA

The viral load was determined by the RealBest HIV-1 kit, quantitative (Vector-Best, Russia), according to the manufacturer's recommendations, using CFX96 thermocycler (BioRad, USA).

### 2.4. Amplification of HIV-1 Gene Fragments and Sequence Analysis

Viral RNAs were extracted from 250 *μ*l of plasma with RealBest DeltaMag kit (Vector-Best, Russia) according to the manufacturer's recommendations. The RNA was utilized for reverse transcription PCR and nested PCR. Amplification was performed using a lyophilized ready-to-use Reverse Transcription Master Mix containing all the components for a single-tube reverse transcription and PCR (Vector-Best, Russia) and an in-house set of primers as described previously [15]. This procedure generates an amplicon of the *pol* gene encoding the protease-reverse transcriptase (PR-RT, 1400 nt), integrase (IN, 960 nt), and a fragment encoding a part of the major envelope protein, *env* (732 nt). For several HIV-1 samples, *gag* (1457 nt) fragment was additionally obtained. After purification, PCR fragments were sequenced by ABI PRISM 3130xl DNA Analyzer (Applied Biosystems, United States).

### 2.5. Sequence Analysis

All original sequence fragments of the *pol* and *env* gene regions were assembled in whole sequences in Sequencher 4.1 software (Gene Codes Corporation, Ann Arbor, MI, United States). The assembled sequences of *pol* fragments (PR-RT and IN) and *env* gene fragments were compared to the corresponding reference sequences of various HIV-1 subtypes and recombinant forms extracted from the Los Alamos HIV Sequence Database (http://www.hiv.lanl.gov) using ClustalW Multiple Alignment and BioEdit software 7.2.5 [25].

MEGA v6.0 was used to construct a phylogenetic tree employing the neighbor-joining method based on the Kimura two-parameter model with 1000 bootstrap replicates based on Kimura's two-parameter model [26]. Statistical significance of phylogenetic tree topologies was estimated using bootstrap analysis. Discordant gene regions or outlier positions in the trees were further analyzed using the jumping profile Hidden Markov Model program (jpHMM; http://jphmm.gobics.de). The similarity between HIV sequences is plotted using SimPlot 3.5.1 software [27] using a 200 nt window with tree construction by the neighbor-joining method applying Kimura's two-parameter substitution model. The possible intertype mosaicisms of URFs were screened using the Recombinant Identification Program (RIP, http://www.hiv.lanl.gov/content/sequence/RIP/RIP.html) and then verified via bootscanning and informative site analysis using the program SimPlot v3.5.1. When determining the most probable geographic origin of novel HIV variants circulating in Krasnoyarsk Krai, each novel URF sequence was aligned with HIV-1 sequences isolated worldwide with the highest identities using the online tool HIV BLAST, available at the LANL HIV database.

The V3 nucleotide sequences were analyzed to estimate the genotypic virus tropism and to confirm the phenotypic coreceptor specificity using the online tool Geno2pheno [coreceptor] (G2P, http://coreceptor.geno2pheno.org) [28] and interpreted using the FPR (false positive rate) cutoff values of 10%. V3 sequences were also submitted to Position-Specific Scoring Matrix (PSSM) (http://indra.mullins.microbiol.washington.edu/webpssm) and the sequence codes were additionally checked for positively charged amino acid residues at 11 and/or 25 codons of the V3 loop [29].

The nucleotide sequences of *pol* gene containing the full-length protease, integrase, and the first 300 codons of reverse transcriptase gene were submitted to Stanford HIV Drug Resistance Database (http://hivdb.stanford.edu, assayed for the presence of mutations determining resistance to protease, reverse transcriptase, and integrase inhibitors (DR mutations)) [30]. The DR mutations were identified based on the WHO-recommended list of mutations for DR surveillance [31].

### 2.6. Statistical Analysis

Analysis of qualitative data was carried out using a modification of Fisher's test for contingency tables of mxn dimension. Evaluation of *p* value was conducted using the Monte Carlo method; the number of simulations during each calculation was one million.

R programming language (version 3.4.0) by Rstudio 1.1.442 was used for statistical analyses of the obtained data.

### 2.7. Nucleotide Sequence Accession Numbers

Study sequences were submitted to GenBank under accession numbers MK002482–MK002621 and MK002622–MK002696.

## 3. Results and Discussion

### 3.1. Demographic Characteristics of the Study Subjects

In total, 162 clinical samples from HIV-infected individuals were collected in Krasnoyarsk Krai from February to December 2017. HIV-specific fragments were successfully obtained and analyzed for 159 patient cases. 81.8% (130/159) of the studied patients were Krasnoyarsk residents, 10.7% (17/159) lived in communities adjacent to the city of Krasnoyarsk, including Achinsk, Sosnovoborsk, and other cities (Krasnoyarsk Krai districts), and 7.6% (12/159) lived in Norilsk (Figure 1).

Women accounted for 47.2% (75/159) with median value of age 35.5 years (range, 21–58); 58.7% (44/75) of them were infected via HS, 13.3% (10/75) via HS with PWID, one woman (1.3%) was infected via homosexual contacts with PWID, 18.7% (14/75) were PWID themselves, and in 8.0% the infection route was not determined. Men accounted for 52.8% (84/159) with the median value of age 38 years (range, 21–51); 27.4% (23/84) were infected via HS and 72.6% (61/84) were PWID. Because of the high stigma in the society, the analyzed sample virtually lacks males with homosexual contacts. Usually, when HIV infection is detected in males with homosexual contacts, they prefer to report the possibility of having been infected with HIV via heterosexual contact. Therefore, the majority of HIV-infected males with homosexual contacts were included in the group of people infected via heterosexual contacts. The date of the last negative and the first positive HIV test of the studied patients was used to estimate a potential period of infection: in case of 63.5% (101/159) the potential period of infection varied from 2015 to 2017, for 25.8% (41/159) from 2009 to 2014, and for 10.7% (17/159) from 1999 to 2008 (Table 1).

### 3.2. High Diversity of HIV-1 Circulating in Krasnoyarsk Krai

**Figure 3:**
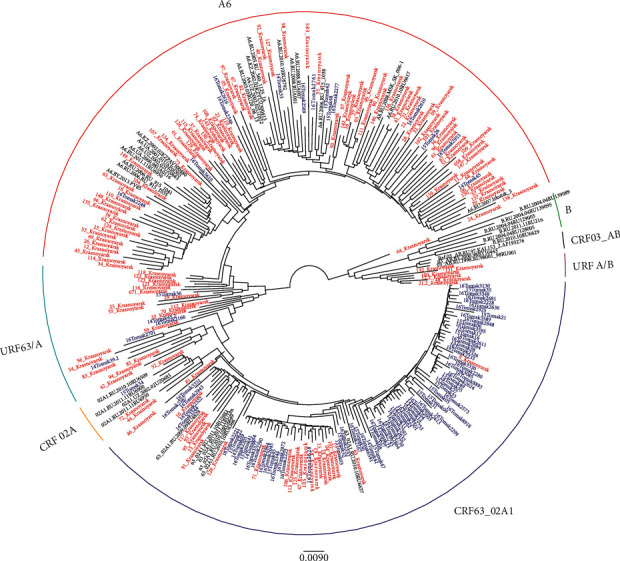
HIV-1 pol PR-RT phylogenetic tree. HIV-1 *pol* PR-RT sequences (∼1.300 nt) circulating in Krasnoyarsk (*n* = 133), Tomsk (*n* = 98), and representative sequences from Novosibirsk. Designations of strains are colored according to the geographic origin of each sequence: red denotes HIV-1 isolated in Krasnoyarsk Krai; blue in Tomsk Oblast; and black HIV-1 reference sequences. HIV-1 isolated from Krasnoyarsk Krai PWID are marked with an asterisk.

For 159 (100%) HIV-1 samples, fragments encoding protease-reverse transcriptase (PR-RT), for 112 (70.4%) integrase (IN), and for 108 (57.9%) fragments encoding V3 region of the major envelope protein (*env*) were obtained. The obtained nucleotide HIV-1 sequences were used for genotyping by constructing phylogenetic trees using the neighbor-joining (NJ) method. Detection of potential recombinant events between virus subtypes was performed using specialized program resources (jpHMM, SimPlot software, RIP). The complex of data obtained during recombination and phylogenetic analyses was used for genotyping of 159 HIV-1 samples (Figure 3).

Among the studied HIV-1, A (A6) subtype specific to Russia was detected in 57.2% and CRF63_02A1 in 12.6%; CRF02_AG_СА_ was genetically similar to HIV-1 circulating in Central Asia in 0.6%. HIV-1 URFs were detected in 29.6% of the cases, including URF63/А (23.9%), URFА/В (4.4%), and URF02/А (1.3%).

It is important to note that Krasnoyarsk subtype A HIV-1 variants are distributed in the phylogenetic tree within subtype A genetic cluster mixing with samples isolated in other Siberian territories. Several HIV-1 subtype A from Krasnoyarsk Krai formed separate subbranches that frequently aggregated viruses isolated from Krasnoyarsk residents PWID and HS patients who probably had sexual contacts with PWID but failed to know about it or to report that their partners might belong to PWID.

Such an example may include separate subbranch of a phylogenetic cluster of subtype A including Krasnoyarsk 53, 62, 135, 39, 66, 34, 43, 114, 12, 26, and 69 (Figure 3). All those HIV variants were isolated from PWID except for Krasnoyarsk 43 isolated from a man with HIV infection detected in 2017 who reported a probable infection route via HS.

In contrast to HIV-1 subtype A, more than half of HIV-1 CRF63_02A1 from Krasnoyarsk Krai formed a separate subbranch (Krasnoyarsk 17, 101, 106, 14, 15, etc.) in a phylogenetic tree constructed for HIV-1 pol region.

Out of 15 HIV-1 in that group, in 7 cases the viruses were isolated from PWID diagnosed in 2014–2017, and 6 HIV-1 were isolated from patients who reported HS with HIV-positive individuals diagnosed in 2015–2017 and two women with undetermined infection route, with HIV newly diagnosed in 2017.

This CRF63_02A1 branch specific to Krasnoyarsk Krai also comprises a substantial number of HIV-1 isolates that were obtained from 2014 to 2016 in Tomsk Oblast from PWID who were using synthetic drugs. These data point to probable HIV-1 CRF63_02A1 transmission from Tomsk Oblast. Among Krasnoyarsk Krai HIV-1, which group together with viruses CRF63_02A1 isolated from Novosibirsk Oblast residents, in one case the virus was detected in PWID who arrived from Kemerovo Oblast adjacent to Novosibirsk Oblast (Krasnoyarsk 10), and in the second case in a man with HIV detected in August 2017, who reported HS (Krasnoyarsk 117).

Out of the remaining eight HIV-1 CRF63_02A1, which did not noticeably group with other HIV-1, seven were isolated from people infected via HS, and one from a patient infected via contact with PWID.

HIV-1 CRF02_AG_СА_ genetic variant was detected in one case (Krasnoyarsk 72) in 37-year-old PWID from the city of Norilsk with HIV detected in 2002.

In 29.6% (47/159) of studied cases, HIV-1 belonged to unique recombinant forms (URF) of the virus including URF63/А (38/159, 23.9%), URFА/В (7/159, 4.4%), and URF02/А (2/159, 1.3%). In all cases, one of the HIV-1 ancestor forms was subtype A (A6).

For URF02/A, the second ancestor form was HIV-1 CRF02_AG_CA_ [32, 33]. In one case, URF02/A virus was isolated from PWID from the city of Norilsk with HIV diagnosed in 2008 (Krasnoyarsk 79), and in the other from a 48-year-old woman from the city of Krasnoyarsk (Krasnoyarsk 44). The woman reported the HS route, and HIV was diagnosed in August 2017.

Out of 48 detected HIV-1 URF, 38 (79.2%) emerged due to the recombination of HIV-1 subtype A and CRF63_02A1 (URF63/А). Out of 38 URF63/А, in 23 (60.5%) HIV-1 recombinant regions were identified in PR-RT region, in 41.7% in the region encoding virus integrase, and in 14.8% in *env* region.

**Figure 4:**
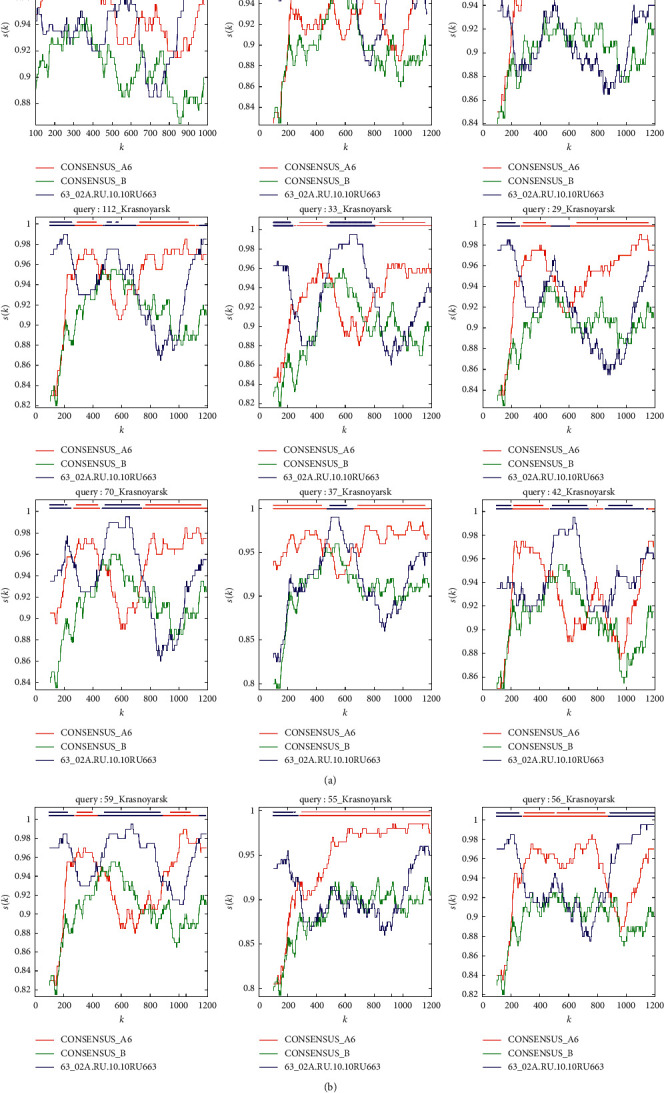
The mosaic pattern of three HIV-1 URF63/A recombinants. Results of recombination breakpoint analyses carried out using Recombinant Identification Program for HIV-1 URF63/A Krasnoyarsk 25, 29, 33, 37, 42, 55, 56, 59, 70, 83, 94, and 112. Bootscanning plots of URF63/A with subtype A6 (consensus_A6, red line), CRF63_02A1 (63_02A.RU.10.10RU663, blue line), and subtype В (consensus_В, green line) as reference genotypes with a window size of 200 nt and confidence threshold 90%.

In most cases, PR-RT regions of HIV-1 URF63/А demonstrated the mosaic structure of genome with significantly differed length and location of interchanged regions identical with HIV-1 subtype A6 and CRF63_02A1 sequences. These data indicate that the origin of HIV-1 URF63/А genetic variants is the result of separate recombination events (Figure 4).

**Figure 5:**
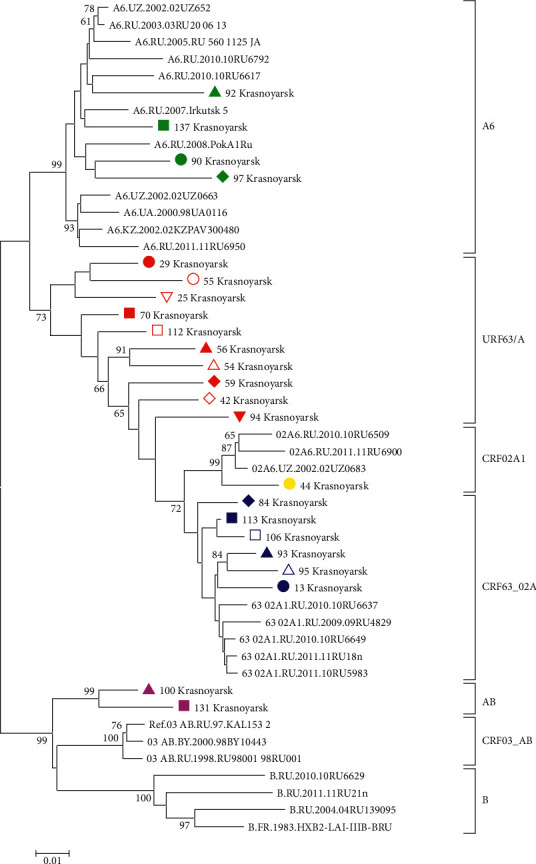
HIV-1 *pol* PR/RT phylogenetic tree. HIV-1 *pol* PR/RT sequences circulating in Krasnoyarsk. Genetic distances were estimated using Kimura's two-parameter model, the clustering of strains was tested with 1000 bootstrap replicates, and the statistical significance of the phylogenetic tree topology was estimated using bootstrap analysis. Different colored figures denote HIV-1 URF grouping with various phylogenetic clusters by PR-RT, IN, and *env* regions.

**Figure 6:**
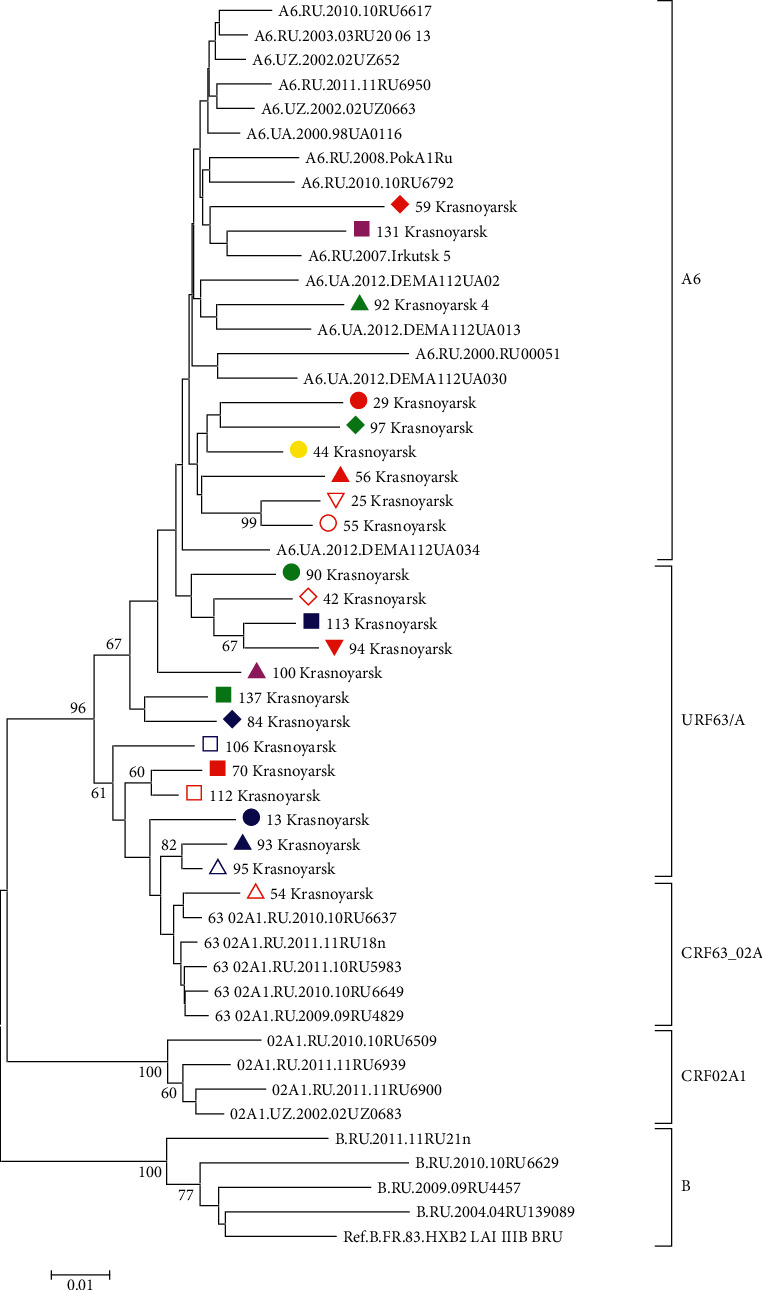
HIV-1 *pol* IN phylogenetic tree. HIV-1 *pol* IN sequences circulating in Krasnoyarsk. Genetic distances were estimated using Kimura's two-parameter model, the clustering of strains was tested with 1000 bootstrap replicates, and the statistical significance of the phylogenetic tree topology was estimated using bootstrap analysis. Different colored figures denote HIV-1 URF grouping with various phylogenetic clusters by PR-RT, IN, and *env* regions.

In some cases, we observed a combination of HIV-1 URF63/A in separate subbranches with a high bootstrap, for instance, HIV-1 samples Krasnoyarsk 54 and 56. However, the recombination analysis of HIV variants in the PR-RT region showed differences in the recombination model, and in the phylogenetic analysis carried out for the virus IN region HIV-1, Krasnoyarsk 54 grouped with HIV-1 CRF63_02A1, and Krasnoyarsk 56 grouped with viruses of subtype A6 (Figures 5 and 6).

The other group of URF63/A with similar PR-RT region includes HIV-1 samples located on a separate subbranch in a phylogenetic cluster of subtype A: Krasnoyarsk 37, 115, and 126.

Out of the three samples, HIV-1 similar to parent variants was reliably detected only for Krasnoyarsk 37, with Krasnoyarsk 37 origin being the result of their recombination. Recombinant analysis using selected reference sequences for HIV-1 Krasnoyarsk 115 and Krasnoyarsk 126 allowed reliable establishment only of the fact that Krasnoyarsk 37, 115, and 126 have a similar profile of recombination; in particular, it is an insertion of sequence that differed from subtype A with approximately the same length and location of recombination regions, which is observed in all HIV-1 samples in this group. For HIV-1 Krasnoyarsk 115 and Krasnoyarsk 126, reliable determination of the origin of the insertion was not possible.

**Figure 7:**
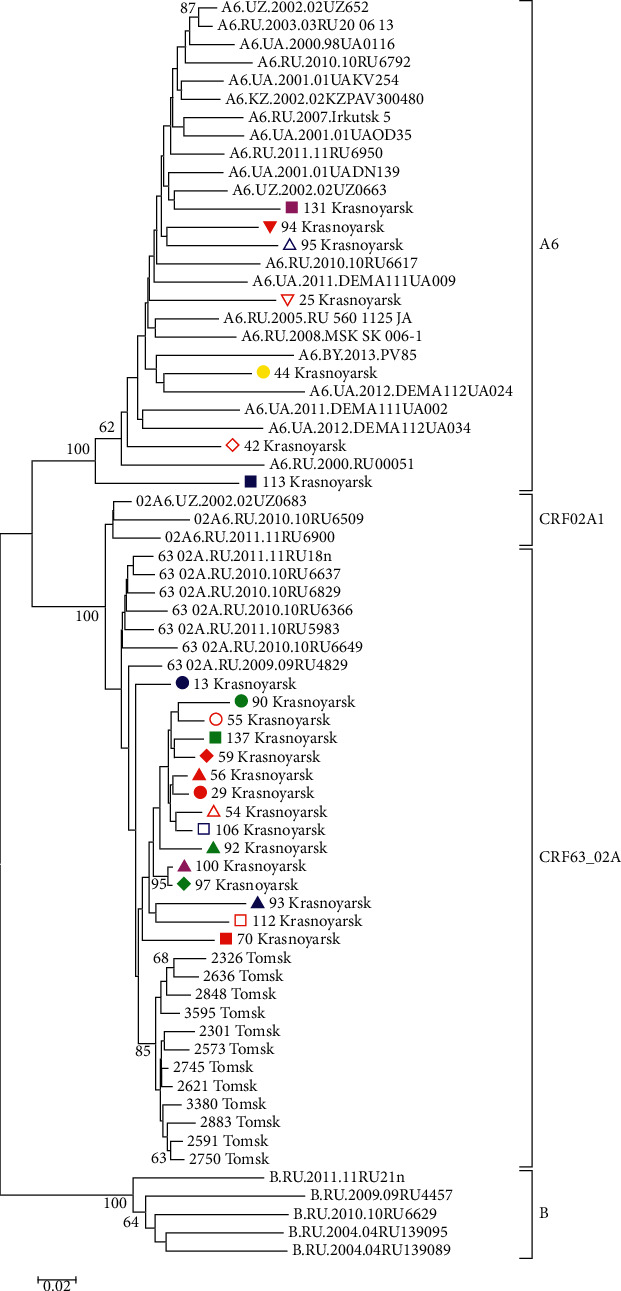
HIV-1 *env* phylogenetic tree. HIV-1 *env* gene fragment sequences circulating in Krasnoyarsk and Tomsk. Genetic distances were estimated using Kimura's two-parameter model, the clustering of strains was tested with 1000 bootstrap replicates, and the statistical significance of the phylogenetic tree topology was estimated using bootstrap analysis. Different colored figures denote HIV-1 URF grouping with various phylogenetic clusters by PR-RT, IN, and *env* regions.

Phylogenetic trees were constructed for all HIV-1 URF with differences in the results of genotyping of virus genome regions encoding PR-RT, IN, and *env* (Figures 5–7). This phylogenetic analysis confirmed differences in clustering with HIV-1 reference sequences.

In 13 cases, HIV-1 URF63/А were isolated from patients infected via HS (nine in 2016-2017 and four in 2014-2015). Two people infected with URF63/А reported HS with PWID (infected in 2013 and 2015) and one patient with homosexual contact with PWID (2017); two women with the undetermined route of infection were diagnosed in 2017.

In 20 cases, HIV-1 URF63/А were isolated from PWID. Three patients from the PWID group were diagnosed in 2014, four in 2014, five in 2016, and seven in 2017.

As distinct from HIV-1 URF63/A heterogenic population, 6 detected URFA/B viruses (Krasnoyarsk 12.2, 21.2, 48, 100, 131, and 132) in phylogenetic tree for *pol* region are part of one branch with a bootstrap of 96 confirming statistically reliable HIV grouping (Figure 3).

**Figure 8:**
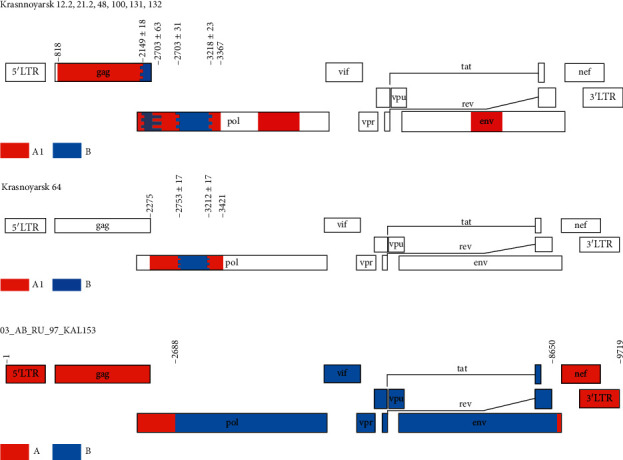
Recombination pattern (according to jpHMM) of the 7 HIV-1 URFA/B isolated in Krasnoyarsk Krai and close HIV-1 CRF03_AB prototype from GenBank (03_AB_RU_97_KAL153_2). The scheme of recombination is presented for 6 HIV-1 since there were no differences detected for them in the studied *gag-pol* region (Krasnoyarsk 12.2, 21.2, 48, 100, 131, and 132).

Recombinant analysis carried out for URFA/B using jpHMM software revealed HIV-1 identical recombination models in Krasnoyarsk 12.2, 21.2, 48, 100, 131, and 132 (Figure 8). Krasnoyarsk 64 demonstrated a similar recombination model with other viruses from that group with several differences in the location of recombination regions in *pol* gene.

Additional sequencing and analysis of *gag* gene for HIV-1 URFA/B made it possible to conclude that those virus variants differed from previously described HIV-1 CRF03_AB [34] since, unlike CRF03_AB viruses, they have an additional insertion of subtype B sequence similar to HIV-1 subtype A *gag* region (Figure 8). All HIV-1 URFA/B demonstrate a similar structure of *gag-pol* region (PR-RT) but differ in IN and *env* sequences: phylogenetic analysis showed that 6 of 7 samples grouped with HIV-1 subtype A (A6) by corresponding genome regions, while Krasnoyarsk 100 sample belonged to URF63/A according to the IN region structure (nucleotide sequence is partly identical to СRF63_02А1 sequence and partly to A6 subtype), and phylogenetic analysis of *env* region of Krasnoyarsk 100 showed that it belongs to HIV-1 CRF63_02A1 phylogenetic cluster (Figures 5–7).

URFA/B was isolated in four Krasnoyarsk patient cases with two of the patients belonging to PWID with HIV detected in 2014 and 2015, and two women infected via HS and diagnosed in August 2017.

Three patients lived in different cities of Krasnoyarsk Krai including two women infected via HS and one PWID man with HIV diagnosed from May 2016 to May 2017. HIV-1 Krasnoyarsk 100, which is a product of recombination of three viruses (URFA/B/6302A1), was isolated from a woman infected via HS with HIV detected in 2017.

### 3.3. Genotypic Analysis of SDRMs and Primary Drug Resistance

Among studied people, 36 underwent ARVT and demonstrated the virological/immunological treatment inefficiency. In 21 of them, HIV-1 was detected with mutations associated with the virus drug resistance development (Table 2). In 16 cases, isolated HIV-1 had mutations that confer resistance to nucleoside reverse transcriptase inhibitors (NRTIs) and nonnucleoside reverse transcriptase inhibitors (NNRTIs), in three cases only to NRTIs and in two cases to NNRTIs.

Among mutations impacting virus sensitivity to NRTIs, we detected M184I, V (11/21, 52.4%), K65R (5/21, 23.8%), T215F (4/21, 19.0%), L74I (2/21, 9.5%), V75I (9.5%), and Y115F (9.5%).

In individual cases, we registered changes M41L, D67N, D67E, D67G, K70E, K70R, Q151M, T215Y, T215S, L210W, and K219E.

The following mutations were associated with the development of resistance to NNRTIs: G190S (9/21, 42.9%), K103N (6/21, 28.6%), Y181C (28.6%), K101E (4/21, 19.0%), A98G (2/21, 9.5%), P225H (9.5%), E138K (9.5%), E138G (4.8%), E138A (4.8%), V179D (4.8%), V179I (4.8%), and V179F (4.8%).

Decrease in sensitivity to protease inhibitors (PIs) was not detected.

Two cases of the high-level HIV-1 resistance development are of particular interest. Krasnoyarsk 16.2 patient: a woman infected via HS with the disease detected in 2014 was under treatment for four years and demonstrated a high level of compliance. Evaluation of the compliance can raise doubts since HIV-1 comprises a set of mutations (M41L, L74I, V75IM, M184IV, T215NSY, Q151M, K103N, E138K, and G190S) causing high-level resistance to all NRTIs, NNRTIs except for tenofovir and etravirine to which low-level resistance was registered.

Krasnoyarsk 16.2 HIV-1 contained mutations, which combines enhanced virus resistance to drugs. M41L and T215Y belong to a group of Thymidine Analog Mutations (TAMs). In combination, M41L plus T215Y confers intermediate/high-level resistance to AZT and d4T and contributes to reduced ddI, ABC, and TDF sensitivity.

Q151M in combination with accessory mutations at positions V75I confers high-level resistance to AZT, ddI, d4T, and ABC and intermediates resistance to TDF, 3TC, and FTC. V75M causes intermediate d4T resistance, low-level ddI resistance, and potentially low-level AZT resistance. L74I causes high-level resistance to ddI and intermediate resistance to ABC.

E138K was detected in combination with M184I. Such a combination is sufficient to cause virological failure on a first-line RPV-containing regimen. High-level resistance to NNRTIs was caused by the presence of K103N and G190S combination.

The second patient Krasnoyarsk 74: a man infected via HS with HIV detected in 2010 was under ARVT for six years; data on compliance evaluation are absent. HIV-1 has a set of mutations (A62V, D67E, T69S_SA, L210W, T215F, K101E, V179F, Y181C, Y188L, V106I, and K238R) resulting in the development of high-level resistance to all NNRTIs and five NRTIs drugs except for emtricitabine and lamivudine, to which intermediate level resistance was detected. In addition to amino acid substitutions in positions connected with the emergence of TAMs, we discovered V179F in combination with Y181C in Krasnoyarsk 74 HIV variant. Such a combination of mutations is associated with high-level ETR and RPV resistance. Additionally, amino acid insertions were identified between codons 67 and 70 (T69S_SA). Together with TAMs, they are associated with high-level resistance to AZT, d4T, ddI, ABC, and TDF and intermediate to 3TC and FTC.

We analyzed primary or transmitted HIV-1 drug resistance for 122 ARVT-naïve patients. In this sample of patients, we detected mutations resulting in the development of different-level HIV-1 resistance in 8.2% (10/122) (Table 3). Transmitted drug resistance was detected in 4 patients infected via HS and 6 PWID patients. As for the virus protease inhibitors, in one case, mutation L76V (0.8%) was found to belong to main PI-resistance mutations. L76V is a nonpolymorphic mutation, which reduces sensitivity to PIs and FPV and NFV; it increases sensitivity to ATV, SQV, and TPV. In the other case, we detected relatively nonpolymorphic mutation M46I. M46I in combination with other PI resistance mutations is associated with reduced sensitivity to each of the PIs except for DRV. Among mutations associated with the resistance to NNRTIs, there were mutations causing significant HIV sensitivity reduction to the following drugs: K103N causing high-level resistance to NVP and EFV (3/122, 2.5%); K101E (1/122, 0.8%) associated with intermediate resistance to NVP and RPV and low-level resistance to EFV and ETR; M230L (0.8%) causing intermediate to high-level resistance to each of the NNRTIs; and V108I (1.6%) resulting in low-level resistance to NVP and potentially low-level resistance to EFV. K103N was found in three women infected via HS with HIV detected from July to November 2017.

Among mutations impacting HIV sensitivity to NTRIs, in individual cases, we found TAMs M41L, D67E, L210W, and T215S. We disregarded A62V since it is widespread among subtype A viruses in the countries of the former Soviet Union [30].

Among other mutations impacting the development of anti-HIV drugs resistance, we detected E138A (5/122, 4.1%), which is a common polymorphic accessory mutation to NNRTIs; it confers a borderline low-level reduction in RPV sensitivity.

In the case of one PWID man with HIV detected in 2016, Major Resistance Mutation associated with the resistance to INSTI (integrase strand transfer inhibitors) E138K (1/122, 0.8%) was found. Alone it does not reduce INSTI sensitivity. When E138K occurs in combination with Q148 mutations, they are associated with high-level resistance to RAL and EVG and moderate reductions in DTG and BIC sensitivity.

### 3.4. HIV-1 *pol* Gene Subtype-Specific Polymorphism and Genotypic Prediction of HIV-1 Coreceptor Usage

A comparative analysis was carried out for *pol* gene sequences encoding protease, reverse transcriptase, and integrase of HIV-1 CRF63_02A1 and subtype A.

For protease region of viruses CRF63_02A1, the presence of amino acid substitutions K20I and I64M was registered in all cases, whereas only one of the mutations, K20I, was found among HIV-1 subtype A, and it was detected in 2.3% of all cases. Substitution I64M is usual for the CRF02_AG genetic variant. Combination of mutations I64M and G17E in HIV protease is believed to be connected with the development of hypersensitivity to NFV, ATV, and IDV. K20I is the consensus amino acid in HIV-1 subtypes G and CRF02_AG.

Mutation V77I in protease common for HIV-1 subtype A in a number of Russian regions was detected only among HIV-1 subtype A in 60.8%. Substitution A62V is specific for reverse transcriptase of HIV subtype A, in the countries of the former Soviet Union. In the studied sample, A62V was registered in 69.1% of HIV subtype A [35–38]. In 100% of studied sequences of reverse transcriptase CRF63_02A1, V60I and D121Y polymorphisms were found that were not associated with the resistance and did not occur among viruses of subtype A.

Among HIV subtype A, 10 variants comprised V90I in the region of reverse transcriptase; furthermore, in 8 of 10 cases, HIV with V90I was detected in patients, which have been treated for more than 5 years with confirmed ARVT inefficiency (virological failure), and only in two instances in naïve patients who were infected 1 and 3 years ago.

Subtype-specific mutations were also detected in the HIV integrase region. In the case of subtype A virus, L74I was detected in 100% of the cases, while among CRF63_02A1 this substitution was detected only in 3%. Alone, L74M/I have minimal, if any, effect on INSTI sensitivity, but in combination with mutation Y143H/R/C L74I, causes reduction of HIV sensitivity to raltegravir [39].

Mutation M50I specific to integrase of HIV-1 CRF63_02A1 spreading in Tomsk Oblast and Novosibirsk Oblast (where it is detected in 85–98%) was detected only in 31.3% among HIV-1 circulating in Krasnoyarsk Krai. In this position in circulating HIV-1 in Krasnoyarsk Krai, the substitution M50T was found most frequently—in 56.3%. Data on the connection between the development of resistance to virus integrase inhibitors and mutation M50T are not available.

M50I is a polymorphic mutation selected *in vitro* by DTG and BIC in combination with R263K. It appears to contribute to reduced DTG sensitivity in combination with R263K [40].

To predict HIV-1 tropism, after two or three re-received nucleotide sequences of V-3 loop for each HIV-1 variant these nucleotide sequences were used for analysis with Geno2pheno [coreceptor] 2.5 software with FPR (false positive rate) equal to 10%.

The tropism was successfully predicted for 108 (67.9%) patients. In 91 cases (84.3%), virus tropism was predicted to coreceptor ССR5, and in 17 to СXCR4 (15.74%). Out of 17 СXCR4-tropic HIV-1, 14 belonged to subtype A, two to URF63/A, and one to CRF63_02A1. In 6 cases, HIV СXCR4-tropism was detected in patients infected in 1999–2013, in 5 cases in 2014–2016, and in 6 patients with HIV infection newly diagnosed in 2017. Four of them had not been tested for HIV earlier; their CD4 value varied from 305 to 603 cells per *μ*l and may indicate earlier period of HIV infection.

## 4. Discussion

Krasnoyarsk Krai is a high-risk territory according to HIV epidemic indices. Despite some stabilization of the epidemic, annual detection of new HIV infection, cases in Krasnoyarsk Krai remain at a high level and increase in HIV incidence is observed among the population. Therefore, molecular epidemiologic analysis of current HIV infection spreading in the region conducted in our study is of current importance for characterization of the epidemic situation.

The previous study of HIV-1 isolated in Krasnoyarsk Krai in 2008 revealed that the epidemic development in the region was accompanied by the distribution of the genetically homogeneous HIV-1 subtype A (A6) population [24].

**Figure 9:**
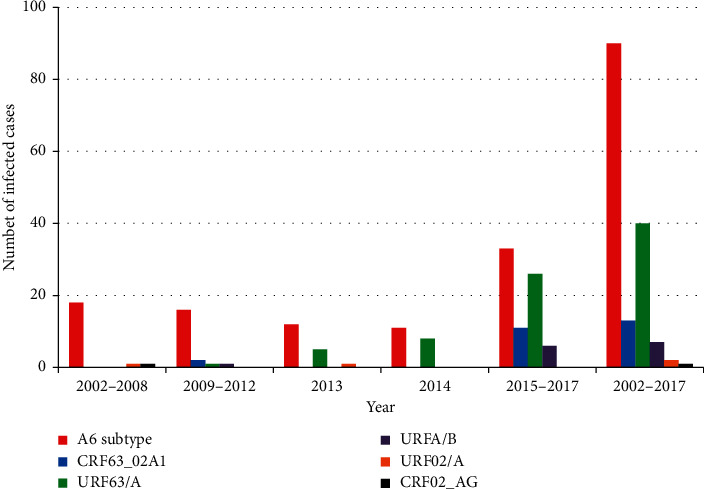
Distribution of HIV-1 genetic variants isolated from Krasnoyarsk Krai individuals taking into account the time of the detection of HIV infection.

A random sample of 159 patients involved in our study includes Krasnoyarsk Krai individuals infected in 1999–2017. Distribution of people included in the sample and living with HIV/AIDS (PLHIV) by gender, age, and infection routes corresponded to current epidemic characteristics. Data on previous negative HIV testing for most of PLHIV allowed the distribution of those patients according to possible infection periods. A group of people infected in 2015–2017 included 101 individuals. Since 2012, an increase in circulating HIV-1 heterogeneity has been registered in Krasnoyarsk Krai both due to the new HIV-1 CRF63_02A1 emergence and distribution in that region and due to the emergence of new unique recombinant HIV-1 forms (Figure 9).

Phylogenetic analysis carried out for HIV-1 isolated in different Siberian regions led to the conclusion that there were at least two independent HIV-1 CRF63_02A1 transmissions to Krasnoyarsk Krai from Tomsk Oblast and Kemerovo Oblast. The complex of data on the detection of phylogenetic clustering and analysis of subtype-specific HIV-1 polymorphism points out that, in Krasnoyarsk Krai, its *region specific* HIV-1 CRF63_02A1 population of variants with specific differences in genome nucleotide sequences has begun to spread, as well.

In 2015–2017, a remarkably high share of people with newly diagnosed HIV infection and with the detection of newly emerged recombinant HIV (38.6%) was registered in the region.

Furthermore, we detected the distribution of two HIV-1 URF of different origins that differed by one of the ancestral forms: URFA/B and URF63/A.

New HIV-1 URFA/B was isolated in 7 cases. In 5 URFA/B cases, we detected a high level of identity for all studied virus regions. One more HIV from that group was identical to 5 HIV-1 URFA/B in *gag-pol* region but was genotyped as URF63/A virus by IN region and was clustered with HIV-1 CRF63_02A1 (Krasnoyarsk 100) in *env* region analysis, which seem to point to the likelihood of the patient reinfection and, consequently, the second recombination between HIV-1 URFA/B and CRF63_02A1 viruses.

HIV-1 URFA/B viruses were isolated in PWID (3), PWID sexual partners (1), and individuals infected via HS (3) living in the city of Krasnoyarsk and other cities of Krasnoyarsk Krai. Considerably restricted (as compared to CRF63_02A1) distribution of this genetic variant suggests that the detected HIV-1 URFA/B, which was first discovered in this study, originated in PWID/or was transmitted by PWID and has been distributed among closed PWID group and their sexual partners.

HIV-1 CRF63_02A1 was isolated for the first time in Krasnoyarsk Krai from women infected via HS in 2010 and 2011. HIV CRF63_02A1 among PWID was detected for the first time only in 2014. In 2015–2017, HIV-1 CRF63_02A1 infection was registered in 15 cases, of which only five patients were PWID.

As HIV-1 heterogeneity in Krasnoyarsk Krai has begun to significantly increase since 2012, we carried out additional analysis of HIV-1 genetic variants registered in 2012–2017 among people practicing risk behavior (HS and PWID).  Table 4 demonstrates that among people infected via HS, an infection with СRF63_02A1 is registered more frequently, while a share of HIV-1 URF63/A in PWID is higher almost by 10% as compared to the HS group.

Distribution of HIV-1 СRF63_02A1 in Krasnoyarsk Krai is registered in different population strata. Due to the high HIV-1 subtype A incidence among Krasnoyarsk Krai population, HIV reinfection is observed including infection with СRF63_02A1 viruses. It is likely that, in PWID, which is the most affected group by HIV infection compared to other population, HIV reinfection occurs more frequently.

It is notable that HIV-1 СRF63_02A1 distribution in Krasnoyarsk Krai significantly differs from other cases of HIV-1 СRF63_02A1 rapid spreading in Novosibirsk Oblast, Tomsk Oblast, Kemerovo Oblast, and Altai Krai that were described earlier [7, 8, 22]. In those Siberian regions, HIV-1 subtype A was predominant up to 2008–2012. HIV-1 СRF63_02A1 was spreading in those regions during local epidemic outbreaks that were accompanied by a multifold increase in morbidity among PWID. After that, further spreading of that HIV genetic variant was registered among HS partners of PWID, and currently, HIV-1 СRF63_02A1 is being registered among the whole population. Among newly diagnosed patients, HIV-1 СRF63_02A1 infection is registered in more than 80–85% of cases in these territories. Kemerovo Oblast is the only exception where the distribution of СRF63_02A1 was detected at the level of 74% already in 2014–2016, and in 21% of the cases, HIV-1 URF63/A infection was registered [7].

In the first years of HIV infection distribution and in 2013–2015, the contribution of PWID transmission to HIV spreading was predominant in Krasnoyarsk Krai; in the course of other periods, HIV was predominantly distributed via HS. This dynamic may be a key factor in the model of epidemic process development [41].

To genotype and detect recombinant HIV-1, we studied not the entire genome, but only three HIV-1 genome regions that probably prevented us from the detection of all recombinant viruses in the analyzed sample.

In support of this hypothesis, we point out that in HIV-1 PR-RT region both “hot” and “cold spots” of recombination were described; a number of studies confirmed that analysis of HIV-1 PR-RT region reveals only about 30% of all recombinant events occurring in HIV-1 genome [42, 43].

When carrying out recombinant analysis, we also encountered some difficulties. In some cases, recombinant analysis of several deduced HIV-1 sequences was not possible due to insufficient availability of publicly accessible close homologous sequences which are necessary for the analysis. To carry out HIV-1 URF recombinant analysis using SimPlot software, in each case, we preliminary performed search for viral sequences with the most close genetic identity to the analyzed HIV sequences from GenBank (using HIV BLAST software (https://www.hiv.lanl.gov/content/sequence/BASIC_BLAST/basic_blast.html)) and among HIV-1 sequences that were obtained from the study of HIV-1 heterogeneity in SFD. Additionally, we carried out the comparable procedure of searching for the most genetically close sequences for each HIV-1 URF recombinant segment without recombination joint; however, in some cases no genetically close sequences were found.

Recombination analysis was complicated by the fact that, in Krasnoyarsk Krai, multiple HIV-1 transmissions from other regions are believed to take place including transmission of CRF63_02A1 and possibly URF63/A. As described previously [7, 8], the considerably heterogeneous HIV-1 CRF63_02A1 and URF63/A population is currently distributed in SFD. An absence of genetically close HIV-1 to be used for comparison (references) does not allow for performing reliable determination of recombinant HIV-1 origin in some cases.

Besides, in a number of cases, it was extremely complicated to identify possible intra- and intersubtypical recombinant events due to a high level of identity of HIV-1 subtype A and CRF63_02A1 sequences (e.g., in the region encoding virus integrase), since it is subtype A that is one of the ancestral viruses for the newly emerged recombinant virus CRF63_02A1 [22].

Significant differences in the distribution of two HIV-1 CRF: CRF63_02А1 and CRF02_AG_СА_ should be noted. Individual cases of HIV-1 CRF02_AG_СА_ registration have been observed in all Russian regions beginning from the 2000s. Usually, HIV-1 CRF02_AG_СА_ infected women who reported HS contacts with people from Central Asia, while men belong to the PWID group.

Even though HIV-1 CRF02_AG_СА_ is continued to be detected in different Russian regions including cases among PWID, this HIV-1 genetic variant and new URF based on this variant have limited distribution in Russia, except for single recombinant HIV-1 CRF63_02А1 variant that emerged from this virus [22, 44].

The analysis of the resistant HIV-1 transmission in Krasnoyarsk Krai revealed infection with HIV-1 carrying mutations associated with the development of the virus resistance to PI, NRTI, and NNRTI in 8.2% of studied cases. Described HIV-1 mutations and the degree of their incidence and analysis of HIV-1 tropism correspond well with data obtained by other researchers for other Russian regions [15, 23, 45–49]. Most frequently detected was substitution K103N (3/10), which is DR-mutation to NNRTI. This mutation is the most widespread among HIV-infected Russian patients undergoing ARVT since the first generation NNRTIs were the most commonly prescribed drugs for a long time and they have the lowest genetic barrier for resistance development [50]. Resistant HIV-1 transmission was detected both among patients infected via HS and among PWID.

## 5. Conclusions

The study of the molecular epidemiological picture of HIV-1 distribution in Krasnoyarsk Krai enabled us for the first time since 2008 to register considerable changes in the features of the developing epidemic. Although subtype A (A6) remains predominant HIV-1 genetic variant, we detected not only transmission and distribution of previously rare HIV-1 genetic variants in Krasnoyarsk Krai but also the emergence of a large variety of new unique recombinant HIV-1 forms, which originated from viruses of subtypes А, В, СRF63_02A1, and CRF02_AG. Our study demonstrated a high frequency of HIV-1 reinfection for newly diagnosed HIV infection cases among Krasnoyarsk Krai population, suggesting that the region population, including PLHIV, is probably insufficiently informed on the negative impact of HIV-1 reinfection on the progress of the disease. Although general coverage of Krasnoyarsk Krai population with HIV testing is high, targeted HIV testing among people practicing risk behavior seems to be insufficient.

High HIV-1 prevalence among Krasnoyarsk Krai population and parallel circulation of the different genetic variants of the virus in certain territories and among risk groups facilitate HIV-1 reinfection including reinfection with genetically different viruses. These conditions are necessary and sufficient for the increase in the frequency of sporadic recombinant events occurrence in the virus genome allowing for HIV-1 rapid evolution. It was shown that random shuffling of HIV genetic material not only increases viral diversity but also enables the virus to evade the response of the human immune system [51]. The increase in circulating HIV-1 genetic diversity is a general characteristic for many current territorial epidemics [43, 49, 52–56].

The uniqueness of the situation (detection of URF HIV-1 in 38.6% of HIV diagnosed people from Krasnoyarsk Krai in 2015–2017) also lies in the fact that monitoring for HIV, in this case, allows for observing the selection process of new viable viruses from a variety of recombinant forms of HIV that occur in the human body. Our studies of HIV-1 isolated from recently infected individuals in Siberian regions carried out in 2015–2018, including this study, for the first time demonstrated the uniquely high contribution of the virus recombinant variability to the increase in heterogeneity of HIV-1 circulating in Russia. This situation is an adverse factor of epidemic development and requires special attention in order to achieve the stabilization of molecular epidemiologic processes in the region.

## Figures and Tables

**Table 1:** Distribution of HIV-infected individuals involved in the study by age, sex, routes of infection, and a possible period of infection with the indication of the detected HIV-1 genetic variants.

	Total number of patients (%)	HIV-1 genotype^†^						*p* value
		A6	CRF63_02A1	CRF02_AG	URF63/A	URFA/B	URF02/A	
*Age (years)*								
20–39	113 (71.1)	63 (55.8)	15 (13.3)	1 (0.9)	28 (24.8)	5 (4.4)	1 (0.9)	0.985
40–49	41 (25.8)	24 (58.5)	5 (12.2)	0	9 (22.0)	2 (4.9)	1 (2.4)	
≥50	5 (3.1)	4 (80.0)	0	0	1 (20.0)	0	0	

*Gender*								
Female	75 (47.2)	42 (56.0)	10 (13.3)	0	17 (22.7)	5 (6.7)	1 (1.3)	0.8
Male	84 (52.8)	49 (58.3)	10 (11.9)	1 (1.2)	21 (25)	2 (2.4)	1 (1.2)	

*Route of infection*								
Heterosexual	67 (42.1)	42 (62.7)	8 (11.9)	0	13 (19.4)	3 (4.5)	1 (1.5)	0.541^††^
Heterosexual with PWID^*∗∗*^	10 (6.3)	4 (40.0)	3 (30.0)	0	2 (20.0)	1 (10.0)	0	
PWID	75 (47.2)	43 (57.3)	7 (9.3)	1 (1.3)	20 (26.7)	3 (4.0)	1 (1.3)	
Homosexual	1 (0.6)	0	0	0	1 (100.0)	0	0	
Not available	6 (3.8)	2 (33.3)	2 (33.3)	0	2 (33.3)	0	0	

*Diagnosis period*								
1999–2008	17 (10.7)	15 (88.2)	0	1 (5.9)	0	0	1 (5.9)	0.005^*∗*^
2009–2012	20 (12.6)	18 (90.0)	2 (10.0)	0	0	0	0	
2013–2014	21 (13.2)	12 (57.1)	3 (9.5)	0	6 (28.6)	1 (4.8)	0	
2015–2017	101 (63.5)	46 (45.5)	15 (15.8)	0	32 (31.7)	6 (5.9)	1 (1)	
1999–2017	159 (100)	91 (57.2)	20 (12.6)	1 (0.6)	38 (23.9)	7 (4.4)	2 (1.3)	

^†^HIV-1 genotype: the result of HIV-1 genotyping obtained after analysis of the complex of data: phylogenetic analysis from RP-RT, IN, and env regions and additional recombination analysis in case of inconsistency of genotyping results; ^††^gradation “homosexual contacts” was excluded from the analysis due to a low number of observations; ^*∗*^*p* < 0.01. ^*∗∗*^PWID: people who inject drugs.

**Table 2:** Characteristics of patients with HIV drug resistance mutations.

Code region of residence		Age (years)	Gender^*∗*^	Transmission risk group^*∗∗*^	HIV genotype^†^	Drug resistance mutations (NRTI)		Extent of ARVT (years NNRTI)	The level of ARVT compliance^*∗∗∗*^	Plasma HIV-1 RNA (copies/ml)^††^	CD4^+^ cell count (cells/mm^3^)^††^
7	Krasn.Krai	40	M	PWID	A6	M184V	K103N	5	High	598	376
9	Krasn. Krai	33	F	Not available	A6	A62V K65R V75I Y115F	G190S	2	High	8310	343
40	Krasnoyarsk	36	F	HTs	A6	M184V	K103N P225H	10	High	1170	598
61	Krasnoyarsk	58	F	HTs	A6	A62V K65R Y115F	V179DY181C	10	Low	2270000	32
73	Norilsk	46	M	HTs	A6	M184V	G190S K101Q	9	High	2703	265
74	Norilsk	43	M	HTs	A6	A62V D67E T69S_SA L210W T215F	K101E V179F Y181C Y188L V106I K238R	6	High	3906	84
75	Norilsk	47	M	HTs with PWID	A6	A62V K65R M184V	10	High	13304	183	
77	Norilsk	35	M	PWID	URF63/A	M184V	K101E E138G G190S	6	Average	27596	54
79	Norilsk	36	M	PWID	URF02/A	M184 T215F	K101E G190S V90I V106I	6	Average	379	135
80	Norilsk	45	M	PWID	A6		K103N	7	Average	1490	322
102	Krasnoyarsk	36	F	HTs with PWID	A6		Y181C	6	Average	7030	23
107	Krasnoyarsk	39	F	HTs	A6	K65R	Y181C G190S K101Q	1	High	535000	239
109	Krasnoyarsk	37	M	PWID	A6	M184V T215F	G190S K101Q	7	High	52500	293
110	Krasn. Krai	38	F	HTs	A6	A62V D67G	E138K	3	Average	49500	473
131	Krasnoyarsk	45	M	PWID	URFA/B	K65R, Y115D	K103N Y181 V179I	1	Average	146000	123
149	Krasnoyarsk	39	F	HTs	A6	T215S		5	High	1640	296
150	Krasnoyarsk	38	M	PWID	A6	M184V	K103N P225H	6	Average	4930	307
583	Norilsk	39	M	PWID	A6	D67N K70R	A98G	10	High	1750	347
658	Norilsk	35	M	PWID	A6	A62V K70E M184V	K101E E138A G190S	8	Average	342143	120
3	Krasnoyarsk	36	M	HTs	А6	L74I M184V K219E	A98GY 181C G190S	6	Average	2600000	121
16.2	Krasn. Krai	33	F	HTs	А6	M41L L74I V75IM M184V T215S Q151M	K103N E138K G190S	4	High	201000	148

^*∗*^Gender: M: male; F: female. ^*∗∗*^transmission risk group: PWID: people who inject drugs; HTs: people infected via heterosexual contacts. ^†^HIV-1 genotype: the result of HIV-1 genotyping obtained after analysis of the complex of data: phylogenetic analysis from RP-RT, IN, and env regions and additional recombination analysis in case of inconsistency of genotyping results. ^*∗∗∗*^The level of ARVT compliance was determined after individual interview with epidemiologist; ^††^plasma HIV-1 RNA and CD4+ cell count detected at the time of sampling of clinical blood samples.

**Table 3:** Demographic characteristics of individuals infected with HIV-1 containing drug resistance mutations.

Code	Region	Age (years)	Gender^*∗*^	Transmission risk group^*∗∗*^	First HIV + test date	HIV-1 genotype^†^	PI resistance mutations	NRTI resistance mutations	NNRTI resistance mutations	Plasma HIV-1 RNA^††^ (copies/ml)	CD4+ cell count^††^ (cells/mm)^3^
11	Krasnoyarsk	37	F	PWID	24.07.2002	A6			K101E, E138A	1354	919
18	Krasnoyarsk	38	M	PWID	20.08.2012	A6		L210W		66400	78
29	Krasnoyarsk	32	M	PWID	28.04.2016	URF63/A		M41ML		284000	246
51	Krasnoyarsk	39	M	PWID	27.10.2015	A6	L76LV			54600	364
98	Krasnoyarsk	35	F	PWID	20.12.2013	A6	M46I	D67E, T215S	V108I	18000	597
124	Krasnoyarsk	35	M	PWID	24.02.2016	A6			M230L	5000	355
44	Krasnoyarsk	48	F	HTs	25.08.2017	URF02/A			K103N	2870	523
95	Krasnoyarsk	40	M	HTs	03.08.2017	URF63/A			V108I, E138A	840000	72
117	Krasnoyarsk	39	M	HTs	31.08.2017	CRF63_02A1			K103N	33000	445
145	Krasnoyarsk	33	F	HTs	01.11.2017	А6			K103N	34800	790

^*∗*^Gender: M: male; F: female. ^*∗∗*^Transmission risk groups: PWID: people who inject drugs; HTs: people infected via heterosexual contacts. ^†^HIV-1 genotype: the result of HIV-1 genotyping obtained after analysis of the complex of data: phylogenetic analysis from RP-RT, IN, and *env* regions and additional recombination analysis in case of inconsistency of genotyping results. ^††^Plasma HIV-1 RNA and CD4+ cell count detected at the time of sampling of clinical blood samples.

**Table 4:** Identification of HIV-1 genetic variants among Krasnoyarsk Krai population with HIV infection detected after 2012 infected via heterosexual contacts and via the use of injectable drugs.

HIV-1 genotype							
Transmission risk group	Total	A6, number (%)	СRF63_02A1, number (%)	URF02_AG, number (%)	URF63/A, number (%)	URFА/В, number (%)	*p* value
PWID	58	28 (48.3)	7 (12.1)	0	20 (34.5)	3 (5.2)	0.631
HTs	61	30 (51.6)	11 (14.5)	1 (1.6)	15 (25.8)	4 (6.5)	
Total	119	58 (48.7)	18 (15.1)	1 (0.8)	35 (29.4)	7 (5.9)	

## Data Availability

The text of the article contains references to the nucleotide sequences of HIV-1 deposited in the Genebank, which are the main primary results of this study. The nucleotide sequences of HIV in the Genebank are publicly available.

## References

[1] (2017). *UNAIDS DATA 2017*.

[2] Beyrer C., Wirtz A. L., O’Hara G., Leon N., Kazatchkine M. (2017). The expanding epidemic of HIV-1 in the Russian federation. *PLoS Medicine*.

[3] Central Research Institute of Epidemiology (2018). *HIV-Infection in Russian Federation in the First Half of 2018*.

[4] Grouping of Territorial Entities of the RF According to Population Size, 2017

[5] Illicit Drug Trends in Central Asia, 54, 2008

[6] Karamov E. V., Gashnikova N. M., Drozdov I. G., Onishchenko G. G. (2009). *Monitoring of HIV Infection in Eurasia. Atlas of the Human Immunodeficiency Virus*.

[7] Gashnikova N. M., Zyryanova D. P., Astakhova E. M. (2017). Predominance of CRF63_02A1 and multiple patterns of unique recombinant forms of CRF63_A1 among individuals with newly diagnosed HIV-1 infection in Kemerovo Oblast, Russia. *Archives of Virology*.

[8] Gashnikova N. M., Bogachev V. V., Baryshev P. B. (2015). A rapid expansion of HIV-1 CRF63_02A1 among newly diagnosed HIV-infected individuals in the Tomsk region, Russia. *AIDS Research and Human Retroviruses*.

[9] United Nations Office on Drugs and Crime (2013). *The Challenge of New Psychoactive Substances: A Report from the Global SMART Programme*.

[10] Bobkov A., Cheingsong-Popov R., Selimova L. (1997). An HIV type 1 epidemic among injecting drug users in the former Soviet union caused by a homogeneous subtype A strain. *AIDS Research and Human Retroviruses*.

[11] Bobkov A., Kazennova E., Khanina T. (2001). An HIV type 1 subtype a strain of low genetic diversity continues to spread among injecting drug users in Russia: study of the new local outbreaks in Moscow and Irkutsk. *AIDS Research and Human Retroviruses*.

[12] Bobkov A. F., Kazennova E. V., Selimova L. M. (2004). Temporal trends in the HIV-1 epidemic in Russia: predominance of subtype A. *Journal of Medical Virology*.

[13] Foley B. T., Leitner T., Paraskevis D., Peeters M. (2016). Primate immunodeficiency virus classification and nomenclature: review. *Infection Genetics and Evolution*.

[14] Thomson M. M., Vinogradova A., Delgado E. (2009). Molecular epidemiology of HIV-1 in St petersburg, Russia: predominance of subtype A, former Soviet union variant, and identification of intrasubtype subclusters. *JAIDS-Journal of Acquired Immune Deficiency Syndromes*.

[15] Gashnikova N. M., Astakhova E. M., Gashnikova M. P. (2016). HIV-1 epidemiology, genetic diversity, and primary drug resistance in the Tyumen Oblast, Russia. *BioMed Research International*.

[16] Kazennova E. V., Laga V. Y., Gromov K. B. (2017). Molecular epidemiological analysis of HIV infection in northern seaports of Russia. *Voprosy Virusologii*.

[17] Tumanov A. S., Kazennova E. V., Gromov K. B. (2017). The molecular epidemiological analysis of HIV infection in Sakhalin Region, Russia. *HIV Infection and Immunosuppressive Disorders*.

[18] Thomson M. M., De Parga E. V., Vinogradova A. (2007). New insights into the origin of the HIV type 1 subtype A epidemic in former Soviet Union’s countries derived from sequence analyses of preepidemically transmitted viruses. *AIDS Research and Human Retroviruses*.

[19] Diez-Fuertes F., Cabello M., Thomson M. M. (2015). Bayesian phylogeographic analyses clarify the origin of the HIV-1 subtype A variant circulating in former Soviet union’s countries. *Infection Genetics and Evolution*.

[20] Aibekova L., Foley B., Hortelano G. (2018). Molecular epidemiology of HIV-1 subtype A in former Soviet union countries. *PLoS One*.

[21] Baryshev P. B., Bogachev V. V., Gashnikova N. M. (2012). Genetic characterization of an isolate of HIV type 1 AG recombinant form circulating in Siberia, Russia. *Archives of Virology*.

[22] Baryshev P. B., Bogachev V. V., Gashnikova N. M. (2014). HIV-1 genetic diversity in Russia: CRF63_02A1, a new HIV type 1 genetic variant spreading in Siberia. *AIDS Research and Human Retroviruses*.

[23] Lebedev A. V., Neshumaev D. A., Kazennova E. M. (2016). Comparative analysis of genetic variants of the HIV-1 circulating in the Irkutsk region in 1999 and 2012. *Voprosy Virusologii*.

[24] Rumyantseva O. A., Olkhovskiy I. A., Malysheva M. A. (2009). Epidemiological networks and drug resistance of HIV type 1 in Krasnoyarsk region, Russia. *AIDS Research and Human Retroviruses*.

[25] Hall T. A. (1999). BioEdit: a user-friendly biological sequence alignment editor and analysis program for Windows 95/98/NT. *Nucleic Acids Symposium Series*.

[26] Tamura K., Stecher G., Peterson D., Filipski A., Kumar S. (2013). MEGA6: molecular evolutionary genetics analysis version 6.0. *Molecular Biology and Evolution*.

[27] Lole K. S., Bollinger R. C., Paranjape R. S. (1999). Full-length human immunodeficiency virus type 1 genomes from subtype C-infected seroconverters in India, with evidence of intersubtype recombination. *Journal of Virology*.

[28] Sing T., Low A. J., Beerenwinkel N. (2007). Predicting HIV coreceptor usage on the basis of genetic and clinical covariates. *Antiviral Therapy*.

[29] Low A. J., Swenson L. C., Harrigan P. R. (2008). HIV coreceptor phenotyping in the clinical setting. *AIDS Reviews*.

[30] Tang M. W., Liu T. F., Shafer R. W. (2012). The HIVdb system for HIV-1 genotypic resistance interpretation. *Intervirology*.

[31] Bennett D. E., Camacho R. J., Otelea D. (2009). Drug resistance mutations for surveillance of transmitted HIV-1 drug-resistance: 2009 update. *PLoS One*.

[32] Lapovok I., Kazennova E., Laga V. (2014). Short communication: molecular epidemiology of HIV type 1 infection in Kazakhstan: CRF02_AG prevalence is increasing in the Southeastern provinces. *AIDS Research and Human Retroviruses*.

[33] Laga V., Lapovok I., Kazennova E. (2015). The genetic variability of HIV-1 in Kyrgyzstan: the spread of CRF02_AG and subtype A1 recombinants. *Journal of HIV and AIDS*.

[34] Liitsola K., Holm K., Bobkov A. (2000). An AB recombinant and its parental HIV type 1 strains in the area of the former Soviet union: low requirements for sequence identity in recombination. *AIDS Research and Human Retroviruses*.

[35] Gashnikova N. M., Totmenin A. V., Sauhat S. R. (2013). Molecular epidemiological characteristics of the HIV 1 transmission in Southern Russia. *HIV Infection and Immunosuppressive Disorders*.

[36] Roudinskii N. I., Sukhanova A. L., Kazennova E. V. (2004). Diversity of human immunodeficiency virus type 1 subtype A and CRF03_AB protease in eastern Europe: selection of the V77I variant and its rapid spread in injecting drug user populations. *Journal of Virology*.

[37] Sukhanova A. L., Rounskii N. I., Bogoslovskaya E. V. (2005). Protease and reverse transcriptase genetic polymorphism in HIV type I subtype A variants predominating in CIS countries. *Molecular Biology*.

[38] Kazennova E. V., Lapovok I. A., Laga V. Y., Vasilyev A. V., Bobkova M. R. (2012). Natural polymorphisms of HIV-1 IDU-A variant pol gene. *HIV Infection and Immunosuppressive Disorders*.

[39] Clotet Sala B., Menéndez-Arias L., Schapiro J. M. (2009). *Guide to Management of HIV Drug Resistance, Antiretrovirals Pharmacokinetics and Viral Hepatitis in HIV Infected Subjects*.

[40] Wares M., Mesplede T., Quashie P. K., Osman N., Han Y. S., Wainberg M. A. (2014). The M50I polymorphic substitution in association with the R263K mutation in HIV-1 subtype B integrase increases drug resistance but does not restore viral replicative fitness. *Retrovirology*.

[41] Mir D., Jung M., Delatorre E., Vidal N., Peeters M., Bello G. (2016). Phylodynamics of the major HIV-1 CRF02_AG African lineages and its global dissemination. *Infection Genetics and Evolution*.

[42] Smyth R. P., Schlub T. E., Grimm A. J. (2014). Identifying recombination hot spots in the HIV-1 genome. *Journal of Virology*.

[43] Yebra G., Frampton D., Cassarino T. G. (2018). A high HIV-1 strain variability in London, UK, revealed by full-genome analysis: results from the ICONIC project. *PLoS One*.

[44] Moskaleychik F. F., Laga V. Y., Delgado E. (2015). Rapid spread of the HIV-1 circular recombinant CRF02-AG in Russia and neighboring countries. *Voprosy Virusologii*.

[45] Kolomeets A. N., Varghese V., Lemey P., Bobkova M. R., Shafer R. W. (2014). A uniquely prevalent nonnucleoside reverse transcriptase inhibitor resistance mutation in Russian subtype A HIV-1 viruses. *AIDS*.

[46] Kolomeets A. N., Kalacheva G. A., Levakhina L. I., Yastrebov V. K., Nurpeisova A. K., Yarusova I. V. (2016). Epidemiological and molecular genetic features of HIV infected persons in the territory of the Siberian federal district. *Medical Almanac*.

[47] Kazennova E., Laga V., Lapovok I. (2014). HIV-1 genetic variants in the Russian far east. *AIDS Research and Human Retroviruses*.

[48] de Parga E. V., Rakhmanova A., Perez-Alvarez L. (2005). Analysis of drug resistance-associated mutations in treatment-naive individuals infected with different genetic forms of HIV-1 circulating in countries of the former Soviet union. *Journal of Medical Virology*.

[49] Lopatukhin A., Kireev D., Kuevda D. (2017). HIV-1 genotyping tropism profile in an HIV-positive population throughout the Russian federation. *Cogent Medicine*.

[50] Tang M. W., Shafer R. W. (2012). HIV-1 antiretroviral resistance: scientific principles and clinical applications. *Drugs*.

[51] Mostowy R., Kouyos R. D., Fouchet D., Bonhoeffer S. (2011). The role of recombination for the coevolutionary dynamics of HIV and the immune response. *PLoS One*.

[52] Chen M., Ma Y. L., Chen H. C. (2018). HIV-1 genetic transmission networks among men who have sex with men in Kunming, China. *PLoS One*.

[53] Tongo M., Dorfman J. R., Martin D. P. (2016). High degree of HIV-1 group M (HIV-1M) genetic diversity within circulating recombinant forms: insight into the early events of HIV-1M evolution. *Journal of Virology*.

[54] Guimaraes M. L., Couto-Fernandez J. C., Eyer-Silva W. D., Teixeira S. L. M., Chequer-Fernandez S. L., Morgado M. G. (2010). Analysis of HIV-1 BF pr/rt recombinant strains from Rio de Janeiro/Brazil reveals multiple unrelated mosaic structures. *Infection Genetics and Evolution*.

[55] Delgado E., Ampofo W. K., Sierra M. (2008). High prevalence of unique recombinant forms of HIV-1 in Ghana: molecular epidemiology from an antiretroviral resistance study. *JAIDS-Journal of Acquired Immune Deficiency Syndromes*.

[56] Delatorre E., Bello G. (2016). Time-scale of minor HIV-1 complex circulating recombinant forms from central and west Africa. *BMC Evolutionary Biology*.

